# Current status of robotic bariatric surgery: a systematic review

**DOI:** 10.1186/1471-2482-13-53

**Published:** 2013-11-07

**Authors:** Roberto Cirocchi, Carlo Boselli, Alberto Santoro, Salvatore Guarino, Piero Covarelli, Claudio Renzi, Chiara Listorti, Stefano Trastulli, Jacopo Desiderio, Andrea Coratti, Giuseppe Noya, Adriano Redler, Amilcare Parisi

**Affiliations:** 1 Department of Digestive and Liver Surgery Unit, St Maria Hospital, Terni, Italy; 2 Department of General and Oncologic Surgery, University of Perugia, Perugia, Italy; 3 Department of Surgical Sciences, “Sapienza” University of Rome, Rome, Italy; 4 Department of General Surgery, Misericordia Hospital, Grosseto, Italy

**Keywords:** Morbid obesity, Bariatric surgery, Robotic, Roux-en-Y gastric bypass, Robot assisted, Gastric bypass, Sleeve gastrectomy, Gastric banding, Duodenal switch, Surgical outcomes, Complications, Anastomotic leak

## Abstract

**Background:**

Bariatric surgery is an effective treatment to obtain weight loss in severely obese patients. The feasibility and safety of bariatric robotic surgery is the topic of this review.

**Methods:**

A search was performed on PubMed, Cochrane Central Register of Controlled Trials, BioMed Central, and Web of Science.

**Results:**

Twenty-two studies were included. Anastomotic leak rate was 8.51% in biliopancreatic diversion. 30-day reoperation rate was 1.14% in Roux-en-Y gastric bypass and 1.16% in sleeve gastrectomy. Major complication rate in Roux-en-Y gastric bypass resulted higher than in sleeve gastrectomy ( 4,26% vs. 1,2%). The mean hospital stay was longer in Roux-en-Y gastric bypass (range 2.6-7.4 days).

**Conclusions:**

The major limitation of our analysis is due to the small number and the low quality of the studies, the small sample size, heterogeneity of the enrolled patients and the lack of data from metabolic and bariatric outcomes. Despite the use of the robot, the majority of these cases are completed with stapled anastomosis. The assumption that robotic surgery is superior in complex cases is not supported by the available present evidence. The major strength of the robotic surgery is strongly facilitating some of the surgical steps (gastro-jejunostomy and jejunojejunostomy anastomosis in the robotic Roux-en-Y gastric bypass or the vertical gastric resection in the robotic sleeve gastrectomy).

## Background

The increased prevalence of obesity in the general population over the past 30 years encouraged researches focused on the development of new treatment options to achieve long-lasting weight loss. Besides noninvasive conservative treatments (e.g. lifestyle modifications, medical treatment, and behavioral therapy), bariatric surgery is now playing an important role in the treatment for obesity. In 1991 the National Institutes of Health Conference Statement on Gastrointestinal Surgery for Severe Obesity developed a consensus stating that bariatric surgery was the most effective treatment for obesity since it is associated with good long-term results in terms of weight loss, glycemic control and decreased mortality [1]. It is widely recognized the growing incidence of obesity and diabetes mellitus as one of the major public burden in the western countries [2]. Current pharmacotherapy provides improvements in only less than 50% of patients with moderate to severe type 2 diabetes mellitus (T2DM). In the United States Roux-en-Y gastric bypass (RYGB) represents the most common bariatric surgical procedure [3]. Adam et al., in their Clinical Controlled Trial, enrolled 1.156 severely obese patients (BMI ≥ 35 kg/m2); they demonstrated that the RYGB surgery induced a significant weight loss, the best health-related quality of life and reduction of major obesity-related complications [4]. The only limit of bariatric surgery is represented by elevate peri-operative morbidity and mortality; in the attempt to reduce and limit this important issue, Minimally Invasive Surgical techniques, initially laparoscopic and then robotic, are becoming more and more frequent [5]. The feasibility and safety are still debated. In 2011 a meta-analysis by Markar highlighted a decreased anastomotic stricture rate in patients undergoing Robotic RYGB (RRYGB) compared to the traditional laparoscopic approach (P = 0.04) [6]. Recently Hagen et al. demonstrated that RRYGB reduced cost of surgery by avoiding the anastomosis-related complications [7]; this was in contrast with the results presented by Scozzari et al. [8]. In their study they concluded that RRYGBP does not associate with significant shorter hospital stay and fewer complications compared to the traditional laparoscopic procedure [7,8]. Recently, a number of studies were published on this subject, for this reason, despite three systematic review were already published [9-11], a new systematic one was needed in order to evaluate the present state of the literature on robotic bariatric surgery.

## Methods

A systematic literature search was performed on PubMed, Cochrane Central Register of Controlled Trials, BioMed Central and on Web of Science from January 2003 to November 2012. The Preferred Reporting Items for Systematic Reviews and Meta-analyses (PRISMA) was followed 005B [12]. Additional file 1. The following *search strategies* were used in PubMed:

– Robot-assisted [All Fields] AND ("bariatric surgery"[MeSH Terms] OR ("bariatric"[All Fields] AND "surgery"[All Fields]) OR "bariatric surgery"[All Fields])

– Robot-assisted [All Fields] AND ("gastric bypass"[MeSH Terms] OR ("gastric"[All Fields] AND "bypass"[All Fields]) OR "gastric bypass"[All Fields] OR "roux en y gastric bypass"[All Fields]) Robot-Assisted[All Fields] AND Sleeve[All Fields] AND ("gastrectomy"[MeSH Terms] OR "gastrectomy"[All Fields])

– ("robotics"[MeSH Terms] OR "robotics"[All Fields] OR "robotic"[All Fields]) AND ("bariatric surgery"[MeSH Terms] OR ("bariatric"[All Fields] AND "surgery"[All Fields]) OR "bariatric surgery"[All Fields])

– ("robotics"[MeSH Terms] OR "robotics"[All Fields] OR "robotic"[All Fields]) AND ("Band"[Journal] OR "band"[All Fields])

All titles and abstracts were assessed to select those focusing on robotic bariatric surgery. Subsequently, the full-text of the selected trials were independently screened by two authors (RCand ST) for eligibility. When there was overlapping between multiple articles published by the same authors and no difference in the examined time, only the most recent trial was enclosed to avoid double counting. The Pubmed function “related articles” and Google Scholar database were used to search further articles. We also searched the online database of relevant high-impact journals such as Surgery for Obesity and Related Diseases, Obesity, Obesity review, International Journal of Obesity, Obesity Surgery and Surgical Endoscopy. The references of the included studies were evaluated for other potential trials. The two screening authors evaluated the eligibility of each trial.

### Inclusion criteria

In this systemic review, we considered both comparative and non-comparative studies, irrespectively of their size, publication status and language, which included patients who underwent robotic bariatric surgery . Comparative studies were included if they focused on selected outcomes of interest, irrespectively of the type of surgical approach used for comparative group (laparoscopic or open).

### Exclusion criteria

Studies in which the outcomes of interest were neither reported nor directly or indirectly inferable.

### Data extraction

#### Primary outcomes

surgical (conversion to open surgery, anastomotic leakage, re-intervention for complications, mortality), bariatric (postoperative Body Mass Index), and metabolic (type 2 DM remission) outcomes were considered.

#### Secondary outcomes

– Surgical ones (major and minor complication rate, pulmonary embolism rate, deep venous thrombosis, 30-days re-admission rate, anastomotic bleeding, gastrojejunostomy anastomotic stricture, post-operative small bowel obstruction, length of hospital stay, operative time).

– metabolic ones (number of patients able to discontinue medical treatment for T2DM at the follow-up and other obesity related morbidities resolution or improvement such as hypertension, sleep apnoea, gastroesophageal reflux and degenerative arthritis).

The included CCT studies were assessed for their methodological quality using the revised and modified grading system of the Scottish Intercollegiate Guidelines Network (SIGN) [13]; the case series assessment was carried out using the checklist for the quality of case series of the National Institute for Health and Clinical Excellence (NICE) [14]. Two authors (RC and CR) independently extracted data for the listed outcomes and assessed the methodological quality of each study, without masking the authors’ names.

## Results

**Figure 1:**
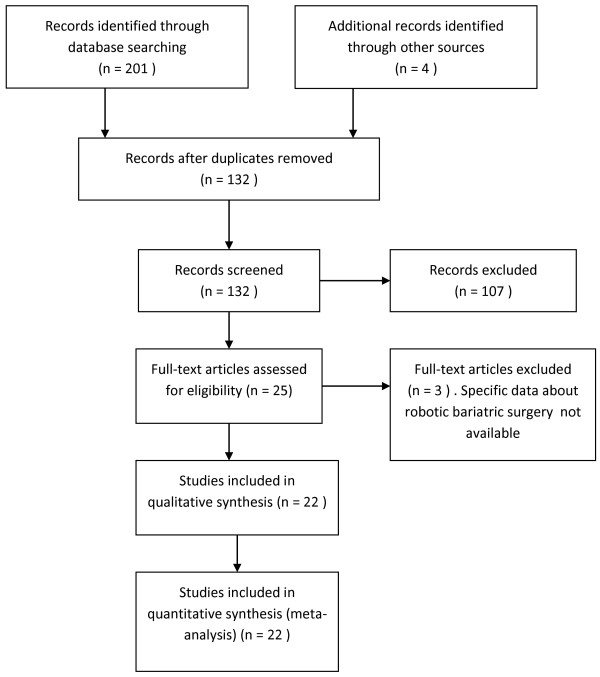
PRISMA flow chart of literature search.

The PRISMA flow chart for systematic review is presented in Figure 1. The initial search produced 132 potentially relevant articles. After the titles and abstracts were screened for relevance, 25 remaining articles were further assessed for eligibility and 3 were excluded; 22 trials whose characteristics are reported in Tables 1 and 2, were included in this systematic review: 1 Randomized Controlled Trial (RCT), 9 Clinical Controlled Trial (CCT) and 12 case series [7,8,15-34]. We excluded the abstract of the largest series trial of robotic-assisted bypass performed in three high-volume centers and presented by Wilson at the American Society for Metabolic & Bariatric Surgery Annual Meeting in San Diego (2012) due to the limited data available in the abstract. [35]. The methodological quality according to the modified grading system of the Scottish Intercollegiate Guidelines Network resulted of fair quality for each of the 10 comparative studies included (mean score 10.3 points) (Table 3). The methodological quality assessment of the case series included proved a fair quality of the selected items evaluated with the NICE checklist (mean score 4.9 points) ( Table 4). The pooled data included 2.781 (patients range per study: 10–1.100 patients) who were planned to receive Robotic bariatric surgical treatment: 2.225 RRYGB, 86 Robotic Sleeve gastrectomy (RSG), 421 silicone adjustable gastric band, 47 bilio-pancreatic diversion with a duodenal switch and 2 implantable gastric stimulator. We excluded from our analysis implantable gastric stimulator (2 patients) and silicone adjustable gastric band reoperation (2 patients). The definition of the robotic approach given in the included studies was very heterogeneous: fully robotic, robotic, robotic-assisted and robot-assisted laparoscopy. The dissection and the resection were also heterogeneous and sequentially combining different approaches: laparoscopic/robotic and only robotic.

**Table 1:** Characteristics of the included studies: setting and technique

Study*	**Years of the study**	**City Nation**	**Type of trial**	**N. of patients**	**Author’s definition of Robotic treatment**	**Type of treatment**	**Type of technique**
Abdalla [15] 2012	2008-2011	São Paulo, Brasil	Case series	27	Robotic assisted gastric band placements	6 Gastric band placements, 5 Vertical gastrectomies and 16 Gastric by-pass in Roux-en-Y	NR^1^
					Robotic assisted vertical gastrectomies		
					Robotic asssisted gastric by-pass in Roux-en-Y		
Buchs [16] 2012	2006-2010	Geneva, Switzerland	Case series	167	Robotic-assisted Roux-en-Y gastric bypass	Roux-en-Y gastric bypass	Laparoscopic-Robotic
Hagen [7] 2012	1997-2010	Geneva, Switzerland	CCT	143	Robotic-assisted Roux-en-Y gastric bypass	Roux-en-Y gastric bypass	Laparoscopic-Robotic
Tieu [17] 2012	2002-2010	Houston, USA	Case series	1100	Robotic-assisted Roux-en-Y gastric bypass	Roux-en-Y gastric bypass	Laparoscopic-Robotic
Vilallonga [18] 2012	2010-2011	Barcelona, Spain	Case series	32	Robot-Assisted Sleeve Gastrectomy	Sleeve Gastrectomy	Fully Robotic
Ayloo [19] 2011	2007 - 2010	Chicago, USA	CCT	30	Robot-Assisted Sleeve Gastrectomy	Sleeve Gastrectomy	Fully Robotic
Diamantis [20] 2011	2008-2009	Athens, Greece	CCT	19	Robotic Sleeve Gastrectomy	Sleeve Gastrectomy	Fully Robotic
Edelson [21] 2011	2006-2009	Philadelphia, USA	CCT	287	Robotic gastric banding	Gastric banding	Fully Robotic
Park [22] 2011	2007-2009	Honolulu, USA	CCT	105	Robotic-assisted Roux-en-Y gastric bypass	Roux-en-Y gastric bypass	NR
Scozzari [8] 2011	2006-2009	Torino, Italy	CCT	110	Robotic-assisted Roux-en-Y gastric bypass	Roux-en-Y gastric bypass	Fully Robotic
Curet [23] 2009	2005	Stanford, USA	CCT	21	Robotic Roux-en-Y gastric bypass	Roux-en-Y gastric bypass	Fully Robotic
Deng [24] 2008	2006-2007	Pasadena, USA	Case series	100	Robotic-assisted Laparoscopic Roux-en-Y gastric bypass	Roux-en-Y gastric bypass	Fully Robotic
Hubens [25] 2008	2004-2006	Antwerpen, The Netherlands	CCT	45	Robotic Roux-en-Y gastric bypass	Roux-en-Y gastric bypass	Fully Robotic
Sudan [26] 2007	NR	Omaha, USA	Case series	47	Robotically assisted biliopancreatic diversion with duodenal switch	Biliopancreatic diversion with duodenal switch	Laparoscopic-Robotic
Parini [27] 2006	2000-2004	Aosta, Italy	Case series	17	Laparoscopic gastric bypass performed with the Da Vinci Intuitive Robotic System	Roux-en-Y gastric bypass	Laparoscopic-Robotic
Mohr [28] 2006	2004-2005	Stanford, USA	Case series	75	Totally Robotic Laparoscopic Roux-en-Y Gastric Bypass	Roux-en-Y gastric bypass	Fully Robotic
Yu [29] 2006	2003-2005	Houston, USA	Case series	100	Robotic assistance for laparoscopic Roux-en-Y gastric bypass	Roux-en-Y gastric bypass	Laparoscopic-Robotic
Ali [30] 2005	2002-2003	Sacramento, USA	Case series	50	Robot-assisted laparoscopic Roux-en-Y gastric bypass	Roux-en-Y gastric bypass	Laparoscopic-Robotic
Artuso [31] 2005	2001-2002	New York, USA	Case series	41	Laparoscopic gastric bypass performed with robotics	Roux-en-Y gastric bypass	Laparoscopic-Robotic
Galvani [32] 2005	2000-2004	Chicago, USA	Case series	140	Robot-assisted surgery	110 Gastric bypass procedures 30 Lap band	Laparoscopic-Robotic
Sanchez [33] 2005	2004-2005	Stanford, USA	RCT	25	Totally robotic laparoscopic Roux-en-Y gastric bypass	Roux-en-Y gastric bypass	Fully Robotic
Muhlmann [34] 2003	NR	Innsbruck, Austria	CCT	10	Robotic-assisted laparoscopic silicone adjustable gastric banding Robotic implantable gastric stimulator	4 silicone adjustable gastric banding	Laparoscopic-Robotic
						2 implantable gastric stimulator	
						4 silicone adjustable gastric banding reoperation	

* Listed in chronological order.

^1^NR: not reported.

**Table 2:** Characteristics of the patients in the included studies

Study*	**Mean preoperative age (years)**	**Mean weight [kg]**	**Mean Body Mass Index [kg/m**^**2**^**]**
Abdalla [15]	NR^1^	NR	NR
Buchs [16]	43	122.8	44
Hagen [7]	42.6	NR	44.5
Tieu [17]	46.9	131.9	47.9
Vilallonga [18]	44.7	NR	48.3
Ayloo [19]	38	152	57
Diamantis [20]	39.4	NR	48.2
Edelson [21]	45	NR	45.4
Park [22]	42.2	NR	46.77
Scozzari [8]	42.6	127.5	46.7
Curet [23]	46.5	NR	45.6
Deng [24]	41.7	NR	48
Hubens [25]	42	NR	44.2
Sudan [26]	38	NR	45
Parini [27]	42.9	NR	50.3
Mohr [28]	44	NR	46.1
Yu [29]	42	NR	50
Ali [30]	42	NR	47
Artuso [31]	42.5	146.2	52.8
Galvani [32]	NR	NR	NR
Sanchez [33]	43.3	NR	45.5
Muhlmann [34]	NR	NR	41.5

*Listed in chronological order.

^1^NR: not reported.

**Table 3:** Evaluation of methodological qualities of comparative included studies

Items/author*	**[**[7]**]**	**[**[19]**]**	**[**[20]**]**	**[**[21]**]**	**[**[22]**]**	**[**[8]**]**	**[**[23]**]**	**[**[25]**]**	**[**[33]**]**	**[**[34]**]**
Inclusion criteria	0	1	0	0	1	0	1	1	1	0
Exclusion criteria	0	0	0	0	0	0	0	0	0	0
Comparable demographics?	1	1	0	1	1	1	1	1	1	1
Could the number of participating centres be determined?	1	1	1	1	1	1	1	1	1	1
Could the number of surgeons who participated be determined?	1	1	1	1	1	0	0	1	1	1
Could the reader determine where the authors were on the learning curve for the reported procedure?	0	0	1	0	0	1	0	1	1	0
Were diagnostic criteria clearly stated for clinical outcomes if required?	1	1	1	1	1	1	1	1	1	1
Was the surgical technique adequately described?	1	1	1	0	0	1	0	1	0	1
Did they try to standardize the surgical technique?	1	0	1	0	0	1	0	1	0	1
Did they try to standardize perioperative care?	0	0	0	0	0	0	0	0	0	0
Was the age and range given for patients in the Robotic group?	1	0	1	1	1	1	0	1	1	1
Did the authors address whether there were any missing data?	1	1	1	1	1	0	1	0	1	0
Was the age and range given for patients in the comparative group?	1	0	0	1	1	1	0	1	1	1
Were patients in each group treated along similar timelines?	1	1	1	1	1	1	1	1	1	0
The patients asking to enter the study, did they actually take part to it?	0	0	0	0	0	0	0	0	1	0
Were drop-out rates stated?	0	0	0	0	0	0	0	0	1	0
Were outcomes clearly defined?	1	1	1	1	1	1	1	1	1	1
Were there blind assessors?	0	0	0	0	0	0	0	0	0	0
Were there standardized assessment tools?	1	0	0	0	0	0	0	0	1	0
Was the analysis by intention to treat?	0	0	0	0	0	0	0	0	1	0
Score	12	9	10	9	10	10	7	12	15	9

Total score, 21; <8, poor quality; 8–14, fair quality; ≥15, good quality.

* Named by reference number and listed in chronological order.

**Table 4:** Evaluation of methodological qualities of observational included studies

Items/author*	**[**[15]**]**	**[**[16]**]**	**[**[17]**]**	**[**[18]**]**	**[**[24]**]**	**[**[26]**]**	**[**[27]**]**	**[**[28]**]**	**[**[29]**]**	**[**[30]**]**	**[**[31]**]**	**[**[32]**]**
Case series collected in more than one centre, i.e. multi-centre study	0	0	1	0	0	0	0	0	0	0	0	0
**Is the hypothesis/aim/objective of the study clearly described?**	1	1	1	1	1	1	1	1	1	1	1	1
Are the inclusion andexclusion criteria **(case definition) clearly reported?**	1	1	1	1	1	1	1	0	1	0	0	0
Is there a clear definition of the outcomes **reported?**	1	1	1	1	1	1	1	1	1	1	1	0
Were data collected prospectively?	0	1	1	0	0	0	0	0	1	1	0	0
**Is there an explicit statement that patients were recruited consecutively?**	0	0	1	1	1	0	1	1	1	0	0	0
Are the main findings of the study clearly **described?**	0	1	1	1	1	1	1	1	1	1	1	0
**Are outcomes stratified? (e.g., by disease stage, abnormal test results, patient characteristics)**	0	0	0	1	1	0	1	1	1	1	1	0
**Total Score**	3	5	7	6	6	4	6	5	7	5	4	1

Yes = 1 No(not reported, not available) = 0.

Total score, 8; ≤3, poor quality; 4–6, fair quality; ≥7, good quality.

* Named by reference number and listed in chronological order.

### Primary outcomes

– Surgical Outcomes: The data listed in Table 5 suggest that robotic bariatric surgery is feasible, regardless of the type of treatment (99.9% in RYGB – 100% in RSG, 100% in silicone adjustable gastric band, 93.62% in biliary pancreatic diversion with duodenal switch). The analysis revealed a very low anastomotic leak rate (0.29% of gastrojejunostomy and 0.05% of jejunojejunostomy in RYGB, 0% in SG, 0.25% in silicone adjustable gastric band, 8.51% in biliary pancreatic diversion with duodenal switch). The 30-day post-operative reoperation rate was very low (1.14% in RYGB and 1.16% in SG) (Table 5). No study reported any case of 30-day postoperative mortality (Table 5).

– Bariatric outcome (postoperative Body Mass Index): only few trials reported the reduced mean BMI after 3 months from the RYGB [7,8,18,24,27,28] and SG [20] (Table 5).

– Metabolic outcomes: none of the studies reported data on the metabolic outcome.

**Table 5:** Primary outcomes

**Study**	**Intraoperative conversions**	**30-day reoperations**	**30-day postoperative mortality**	**Mean body mass index 3 months after surgery**
Abdalla [15]	0	1	0	NR^1^
Buchs [16]	2	2	0	NR
Hagen [7]	2	1	0	44.5
Tieu [17]	0	NR	0	39.8
Vilallonga [18]	0	0	0	NR
Ayloo [19]	0	1	0	NR
Diamantis [20]	0	0	0	reduced of 31.3%
Edelson* [21]	0	11	0	NR
Park [22]	1	1	0	NR
Scozzari [8]	0	2	0	reduced of 33.6%
Curet [23]	NR	NR	0	NR
Deng [24]	NR	0	0	17.5%
Hubens [25]	9	2	0	NR
Sudan [26]	3	NR	0	NR
Parini [27]	0	0	0	39.07
Mohr [28]	4	NR	0	reduced of 48%
Yu [29]	0	2	0	NR
Ali [30]	NR	NR	NR	NR
Artuso [31]	NR	NR	NR	NR
Galvani [32]	NR	NR	NR	NR
Sanchez [33]	1	NR	NR	NR
Muhlman [34]	NR	NR	0	NR

* Listed in chronological order.

^1^NR: not reported.

### Secondary outcomes

– Surgical Outcomes: major complication rates were 4,26% in RYGB and 1,2% in SG; minor complication rates were 1% in RYGB and 0% in SG; Pulmonary embolism rates were 0,71% in RYGB 0% in RSG; deep venous thrombosis rates were 0,37% in RYGB and 0% in SG; 30-day re-admission rates were 4,84% in RYGB and 0% in SG (Table 6). 15 cases of anastomotic bleeding were reported over a total of 1.873 RYGB while none were reported in SG (Tables 7, 8). Gastrojejunostomy anastomotic stricture rate was 1,23% in RYGB. Post-operative small bowel obstruction rates were 1,17% in Roux-en-Y gastric bypass and 0% in sleeve gastrectomy (Tables 7, 8). The mean hospital stay ranged between 2.72 and 7.4 days in RYGB and between 2.6 and 4 days in SG (Table 9). The mean operative time ranged between 130.8 and 295 min. in RYGB and between 95 and 135 min. in SG (Table 9).

– Metabolic outcomes: none of the studies reported this outcome.

**Table 6:** postoperative complications and 30 day readmission in the included studies

Study*	**30-day major complications**	**30-day minor complications**	**Pulmonary embolism**	**Deep venous thrombosis**	**Readmissions in the first 30 postoperative days**
Abdalla [15]	1	5	0	0	NR^1^
Buchs [16]	24 (not classified)		7	2	2
Hagen [7]	23 (not classified)		NR	NR	NR
Tieu [17]	45	102	2	3	67
Vilallonga [18]	1 case	NR	NR	NR	NR
Ayloo [19]	NR		NR	NR	0
Diamantis [20]	0 (not classified)		0	0	0
Edelson [21]	NR		NR	NR	NR
Park [22]	10 (not classified)		0	0	0
Scozzari [8]	4	14	1	NR	NR
Curet [23]	3 (not classified)		NR	NR	NR
Deng [24]	4	7	NR	NR	3
Hubens [25]	NR	NR	NR	NR	NR
Sudan [26]	NR	NR	NR	NR	NR
Parini [27]	0	0	0	0	0
Mohr [28]	6	7	NR	NR	NR
Yu [29]	NR	NR	1	NR	NR
Ali [30]	NR	NR	NR	NR	NR
Artuso [31]	NR	NR	NR	NR	NR
Galvani [32]	NR	NR	NR	NR	NR
Sanchez [33]	NR	NR	NR	NR	NR
Muhlmann [34]	NR	NR	NR	NR	NR

* Listed in chronological order.

^1^NR: not reported.

**Table 7:** Surgical complications after gastric bypass

Study*	**Post-operative anastomotic leak**		**Anastomotic stricture Gastro-jejunostomy**	**Anastomotic bleeding**	**Post-operative bowel obstruction**
	**g-j**	**j-j**			
Abdalla [15]	0	0	0	0	0
Buchs [16]	0	0	3	NR^3^	1
Hagen [7]	0	0	0	3	NR
Tieu [17]	1	1	7	9	19
Park [22]	2	0	2	0	0
Scozzari [8]	2	0	3	0	1
Curet [23]	0	0	0	0	0
Deng [24]	1	0	4	3	0
Hubens [25]	0	0	2	0	1
Parini [27]	0	0	0	0	0
Mohr [28]	0	0	2	0	0
Yu [29]	0	0	2	0	0
Ali [30]	NR	NR	NR	NR	NR
Artuso [31]	1	0	0	0	0
Galvani [32]	NR	NR	NR	NR	NR
Sanchez [33]	NR	NR	NR	NR	NR

* Listed in chronological order.

^3^NR: not reported.

**Table 8:** Surgical complications after sleeve gastrectomy and duodenal switch

**Surgical treatment**	**Study**	**Suture leak**	**Suture stricture**	**Suture bleeding**	**Bowel obstruction/internal hernia**
Sleeve gastrectomy	Vilallonga [18]	0	0	0	0
	Ayloo [19]	0	1	0	0
	Diamantis [20]	0	0	0	0
Duodenal switch	Sudan [26]	4	0	0	0

**Table 9:** Secondary outcomes: mean operative time and mean hospital stay

Study*	**Mean operative time (min.)**	**Length of hospital stay Mean ± Standard deviation (days)**
Abdalla [15]	NR^1^	NR
Buchs [16]	295.2	7.2 ± 2.5
Hagen [7]	293	7.4 ± 2.6
Tieu [17]	155	NR
Vilallonga [18]	130.2	NR
Ayloo [19]	135	2.6
Diamantis [20]	95.5	4
Edelson [21]	91.5	1.3
Park [22]	169	3.41 ± 7.03
Scozzari [8]	247.5	7.8
Curet [23]	181.7	3
Deng [24]	186.3	1.5
Hubens [25]	242.2	4.7
Sudan [26]	514	NR
Parini [27]	201	9
Mohr [28]	140	2.9
Yu [29]	254	NR
Ali [30]	NR	NR
Artuso [31]	289	4.6
Galvani [32]	NR	NR
Sanchez [33]	130.8	2.72
Muhlman [34]	137	3

*Listed in chronological order.

^1^NR: not reported.

## Discussion

The present study revealed slightly different outcomes and complication rates between the traditional laparoscopic approach and the robotic one. Data from a RCT collected after laparoscopic gastric bypass showed that 1-year mortality is about 0.9%, Major perioperative complications (hemorrhage, obstruction, internal herniation, or renal insufficiency) occur in 6.3% of patients and late (> 30 days postoperatively) major complications, more often stenosis or strictures, in 26.1% of them [36]. The results of this review demonstrated that the robotic approach is safe and feasible in all types of bariatric surgical procedures. The overall post-operative complication rate was very low; in particular the anastomotic leak rate (gastro-jejunostomy and jejuno-jejunostomy in RYGB) and the gastric staple line leak rate were very low and no deaths were reported. The analysis of these selected trials on the robotic bariatric surgery did not show any significant results about the bariatric and the metabolic outcomes. Our results were in line with the ones presented in 2012 by Wilson et al. at the Annual Meeting of the American Society for Metabolic & Bariatric Surgery in San Diego [35]. In this trial the authors enrolled 1,695 patients undergoing robotic-assisted RYGB surgery; the post-operative complications were 17 bowel obstructions, 5 wound infections and 18 cases of bleeding. The hospital readmissions rate was 4.8% and re-intervention rate was 2.7%. Leak and anastomotic stricture rates were very low: 0.3% and 0.2% respectively. No death was reported. “This report of the largest series of robotic-assisted bypasses from three high-volume centers reveals very low complication rates in the first 30 days. It reveals zero 30-day mortality, an exceptionally low leak rate, and provides strong evidence that Robot-Assisted RYGB (RARYGB) has extremely safe and reproducible outcomes” [35]. Robotic surgery allowed the reduction of the postoperative complications, especially the anastomotic dehiscence. The low anastomotic leak rate after robotic bypass can be partially explained by the improved accuracy and precision of intracorporeal suturing compared to the traditional laparoscopic approach. 5 cm proximal to the anastomosis, an antireflux longitudinal valve is fashioned with suture stitches 1 cm apart from each other. In USA the RARYGB represent the first line choice of bariatric surgery, but because of its “complexity”, this operation has always been challenged by alternative surgical procedures [37].

Safety of gastric bypass was demonstrated and its effectiveness in the long term weight loss maintenance as well, nevertheless it associates with a long learning curve and it is not free from

complications [38,39]. Kim et al. concluded that the use of the robot is ideal in performing RYGB [40]. This technique associates with shorter learning curve especially in performing delicate and precise manoeuvres such as fine dissections and suturing. Indeed it is widely recognized that robotic bariatric surgery, in particular RRYGB, has a steeper learning curve than laparoscopic approach and 20 cases may be enough to pass the basic learning phase [41]. Moreover this technique, unlike laparoscopic surgery, can be used in high-risk obese patients with difficult anatomy without compromising the surgical performance and outcomes [40]. The best results derived from the RSG that showed even fewer postoperative complications and no mortality, but the use of the robot in performing sleeve gastrectomy is still controversial, and not largely spread among bariatric surgeons yet. Recently few case series on this technique were published [19,20]. Robotic approach was demonstrated associating with shorter learning curve compared to the traditional laparoscopic techniques [37]. A poster presented in by Miller et al. at the annual meeting of the Society of American Gastrointestinal and Endoscopic Surgeons (SAGES) compared 277 laparoscopic sleeve Gastrectomy (LSG) to 40 RSG. The mean operative time was significantly shorted (91 minutes) for LSG compared to the RSG (113 minutes) (p-0.002) No differences were revealed in overall mean hospital stay (2.4 days in the LSG group and 2.5 in the RSG group) (p = 0.86). The overall mean 90-day complication rate requiring readmission was significantly lower in patients who had undergone RSG (12.3% in the LSG group and 5% in RSG group) (p = <.001) [42].

## Conclusion

Robotic assistance is used in a small percentage of bariatric procedures in the US. The major limitation of our analysis is the lack of studies and their low quality, small sample size,, heterogeneity of enrolled patients and the lack of data from metabolic and bariatric outcomes. Despite the use of the robot, the majority of these cases are completed with stapled anastomosis. The assumption that robotic surgery is superior in complex cases is not supported from actual evidence. According to our experience the major strength of the robotic surgery is strongly facilitating some of the surgical steps (gastro-jejunostomy and jejunojejunostomy anastomosis in the robotic Roux-en-Y gastric bypass or the vertical gastric resection in the robotic sleeve gastrectomy). According to our experience the major disadvantage of the robotic bariatric surgery “still remains the high operational and acquisition cost of the system” [37].

## Competing interest

The Authors all report no conflicts of interest. Furthermore for the writing of this paper the Authors didn’t benefit of any source of funding.

## Authors’ contributions

RC designed and concepted the manuscript, performed the interpretation of data, drafted and revised critically the manuscript. CB designed, concepted and revised critically the manuscript. AS analyzed the data and revised the manuscript. SG designed, drafted and revised the paper. PC took part to the interpretation of data and revised the manuscript. CR was involved in the acquisition of data, in their analysis and in drafting the manuscript. CL contributed to the acquisition of data and she took part in drafting the manuscript. ST performed the interpretation of data, drafted and revised critically the manuscript. JD took part to the acquisition of data, in their analysis and in drafting the manuscript. AC was involved in the interpretation of data and revised the manuscript. GN designed, concepted and revised critically the manuscript. AR designed, concepted and revised critically the manuscript. AP concepted and revised critically the manuscript.All authors read and approved the final manuscript and they agree to be accountable for all aspects of the work in ensuring that questions related to the accuracy or integrity of any part of the work are appropriately investigated and resolved.

## Pre-publication history

The pre-publication history for this paper can be accessed here:

http://www.biomedcentral.com/1471-2482/13/53/prepub

## Supplementary Material

Additional file 1 PRISMA Checklist.doc. Click here for file
